# Housing equity for health equity: a rights-based approach to the control of Lassa fever in post-war Sierra Leone

**DOI:** 10.1186/1472-698X-13-2

**Published:** 2013-01-02

**Authors:** J Daniel Kelly, M Bailor Barrie, Rachel A Ross, Brian A Temple, Lina M Moses, Daniel G Bausch

**Affiliations:** 1 Baylor College of Medicine, Houston, TX, USA; 2 College of Medicine and Allied Health Sciences, Freetown, Sierra Leone; 3 Massachusetts General Hospital, Boston, MA, USA; 4 Texas Tech University Health Sciences Center, Lubbock, TX, USA; 5 Department of Tropical Medicine, Tulane School of Public Health and Tropical Medicine, 1430 Tulane Avenue SL-17, New Orleans, LA, 70112, USA; 6 Doctors for Global Health, Atlanta, GA, USA

**Keywords:** Lassa fever, Arenavirus, *Mastomys natalensis*, Housing equity, Health equity, Public health, Global health

## Abstract

Poor quality housing is an infringement on the rights of all humans to a standard of living adequate for health. Among the many vulnerabilities of those without adequate shelter is the risk of disease spread by rodents and other pests. One such disease is Lassa fever, an acute and sometimes severe viral hemorrhagic illness endemic in West Africa. Lassa virus is maintained in the rodent *Mastomys natalensis*, commonly known as the “multimammate rat,” which frequently invades the domestic environment, putting humans at risk of Lassa fever. The highest reported incidence of Lassa fever in the world is consistently in the Kenema District of Sierra Leone, a region that was at the center of Sierra Leone’s civil war in which tens of thousands of lives were lost and hundreds of thousands of dwellings destroyed. Despite the end of the war in 2002, most of Kenema’s population still lives in inadequate housing that puts them at risk of rodent invasion and Lassa fever. Furthermore, despite years of health education and village hygiene campaigns, the incidence of Lassa fever in Kenema District appears to be increasing. We focus on Lassa fever as a matter of human rights, proposing a strategy to improve housing quality, and discuss how housing equity has the potential to improve health equity and ultimately economic productivity in Sierra Leone. The manuscript is designed to spur discussion and action towards provision of housing and prevention of disease in one of the world’s most vulnerable populations.

## Introduction

Poor quality housing is an infringement on the rights of all humans to a standard of living adequate for good health. When invoking Article 25 of the United Nations Declaration of Human Rights, which has been adopted by the United Nations in 1948, there is a tendency to focus on medical care as a means of improving health and well-being, but housing is also included in the article: “Everyone has the right to a standard of living adequate for the health and well-being of himself and of his family, including food, clothing, *housing*, and medical care and necessary social services…” Among the many vulnerabilities of those without adequate shelter is the risk of disease spread by rodents and other pests who may gain entry into houses. One such disease is Lassa fever.

**Figure 1:**
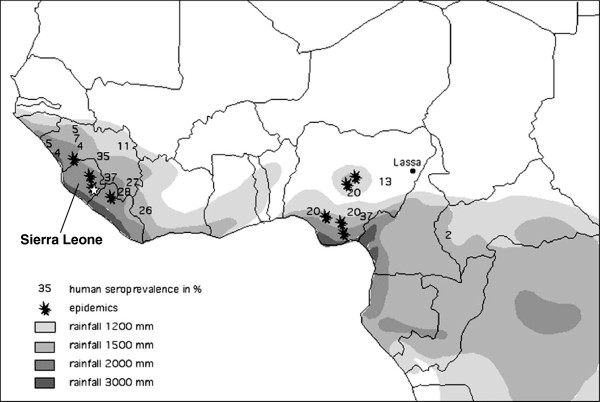
**West and Central Africa mean annual rainfall (1951–1989), Lassa fever nosocomial outbreaks (stars) and human seroprevalence (numbers in %).** The Kenema District of Sierra Leone is indicated by a white star. Adapted from Fichet-Calvet and Rogers, 2009 [4].

**Figure 2:**
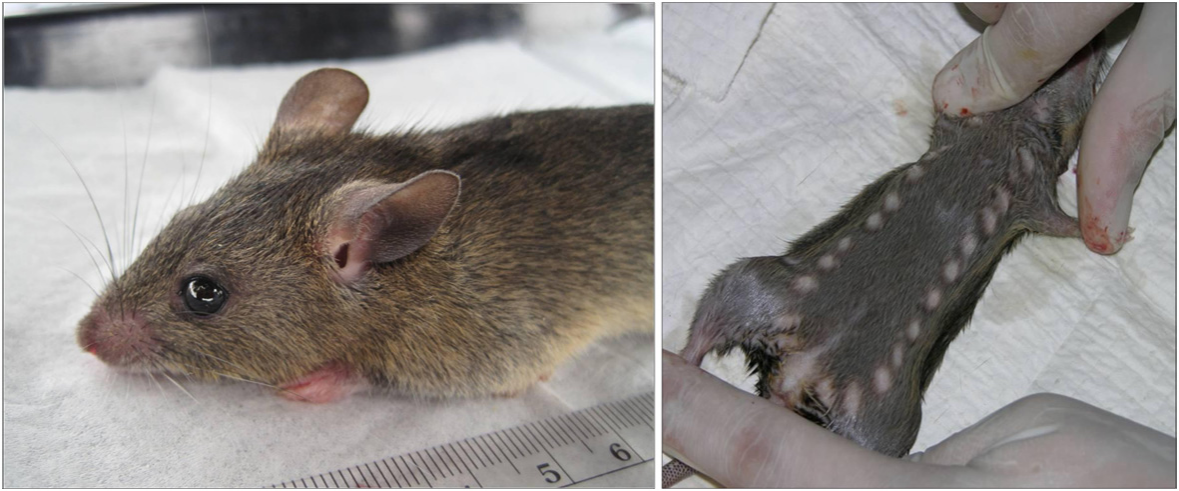
***Mastomys natalensis*, the reservoir of Lassa virus.** The animal is commonly known as the “multimammate rat” due to the female’s multiple and prominent mammary glands, as seen in the right-hand panel. Photos: L. Moses and D. Bausch.

Lassa fever is an acute and sometimes severe viral hemorrhagic illness caused by Lassa virus [1]. The disease is endemic in West Africa, with estimates of up to 300,000 persons infected and 5,000 deaths annually across the region and a population at risk numbering in the millions (Figure 1) [2-5]. Lassa virus is maintained in nature in the rodent *Mastomys natalensis*, commonly known as the “multimammate rat” (Figure 2) [6]. Multimammate rats are also reservoirs of the causative agents of leptospirosis, plague, and leishmaniasis [7-11]. In endemic regions up to 80% of all rodents caught in houses are multimammate rats with the prevalence of Lassa virus infection in these animals ranging from 6-50% [2,12-15]. Transmission to humans occurs primarily via exposure to rodent excreta, as well as when rodents are caught and prepared as food [2,12,16]. Secondary transmission between humans occurs through direct contact with infected blood and bodily fluids, usually in the process of caring for a sick family member or in the nosocomial setting.

Multimammate rats are ubiquitous in grasslands and cleared forest across sub-Saharan Africa. The rodent commonly invades the domestic environment, where transmission to humans is thought to most frequently occur [2,12]. The risk of contracting Lassa virus is thus directly linked to factors that lead to increased rodent infestation in and around the home, which may put housewives, children, and others who spend a lot of time at home at risk.

### Housing quality and Lassa fever in Sierra Leone

The Eastern Province of Sierra Leone, especially Kenema District, has one of the highest reported incidence of Lassa fever in the world [2,17,18]. This area, rich in diamonds and other mineral wealth, was at the center of Sierra Leone’s civil war that lasted from 1991–2002, with tens of thousands lives lost and millions displaced. Countless villages and over 350,000 dwellings were destroyed [19]. Despite the end of the war, about 70 percent of Sierra Leone’s population still lives in poverty. The World Bank ranks Sierra Leone’s per capita GDP at 185 out of the world’s 190 countries, with a United Nations Development Programe (UNDP) Human Development Index rank of 180 out of 187 countries.

**Figure 3:**
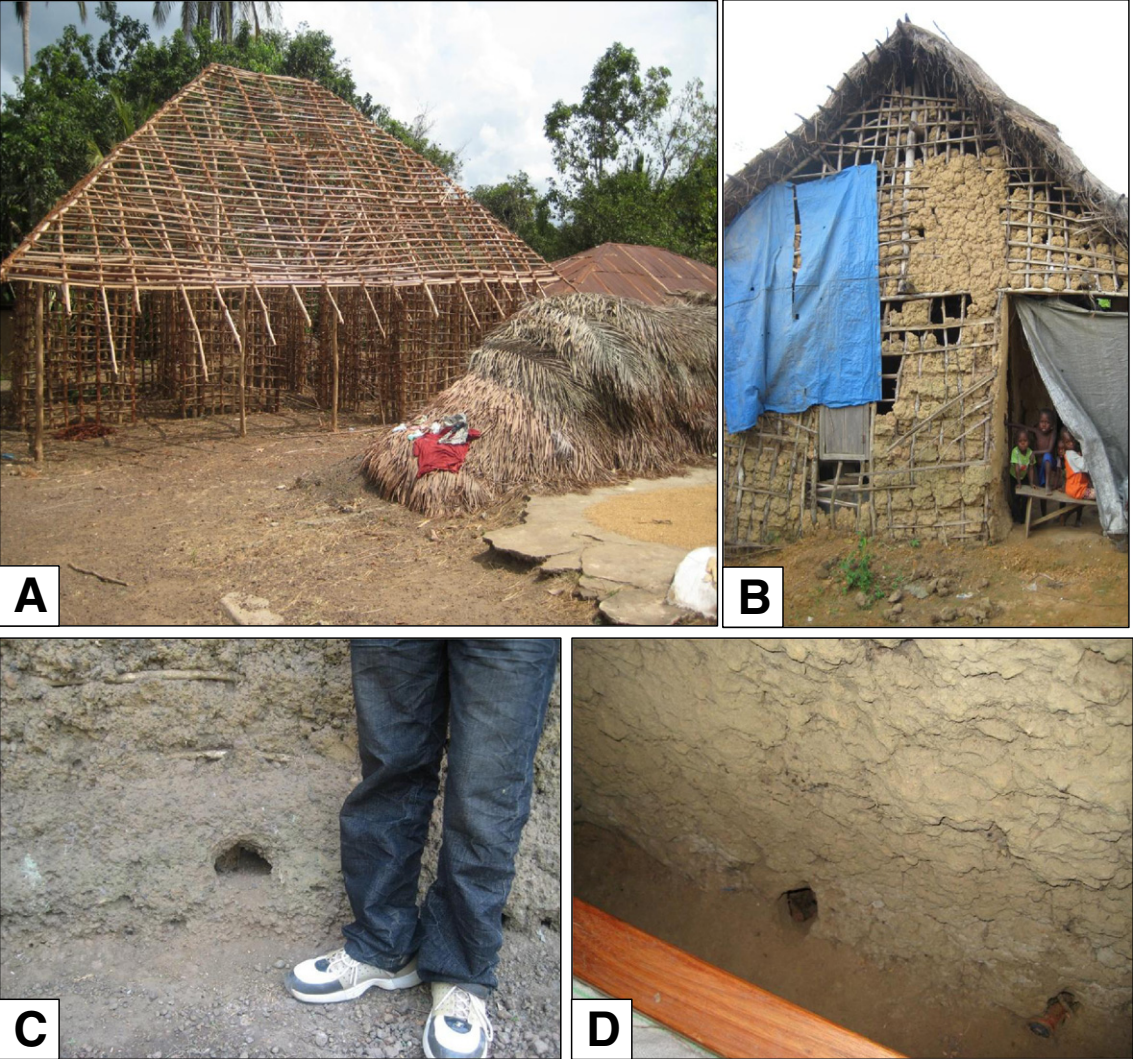
**Typical building materials and homes made of mud and stick in rural areas of Kenema District (Panels A and B).** A blue tarp provided by the United Nations High Commission for Refugees forms part of the wall in the home in Panel B. Rodent burrows can be seen in the mud foundation in the exterior (Panel **C**) and interior (Panel **D**) of houses. Photos: L. Moses.

Not surprisingly, given the devastation endured in Kenema, as recently as 2007, 10 out of the District’s 17 Chiefdoms were considered to have “poor or very poor” housing quality based on a multidimensional indicator composite index of core housing components, including material, roof, foundation, and repair needs [20]. Over 70% of houses are constructed of unbaked mud brick or mud and wattle with 26% roofed with thatch or other poor quality roofing material [20]. Many people continue to live in houses initially intended to provide temporary shelter during the war. The site of roofs made from blue tarps commonly provided to refugees and internally displaced persons by the United Nations High Commission for Refugees is not rare (Figure 3).

In 2007, Bonner *et al.* investigated the relationship between housing quality and risk of Lassa fever in refugee camps in Kenema District [21]. Houses in the camps, constructed primarily of mud bricks and which had existed for years, were similar to those elsewhere in rural Sierra Leone. Houses where cases of Lassa fever had been reported had significantly worse housing quality and external hygiene and more rodent burrows (Figure 3). Another study conducted in eastern Sierra Leone showed that the presence of visible rodent burrows correlated directly with the abundance of multimammate rats in the home [22]. Houses with mud brick or mud and wattle walls were also found to be almost 10 times more likely to have multimammate rat infestation than houses with cement walls (L. Moses, unpublished data).

### Public health interventions for Lassa fever in Kenema District

Control programs for Lassa fever and other rodent-borne diseases are generally comprised of education and awareness raising campaigns to improve “village hygiene,” which include measures such as eliminating unprotected storage of garbage and foodstuffs, clearing vegetation adjacent to houses, and plugging holes that allow rodents entry. Keeping cats as rodent predators is also often suggested. Rodent trapping may help to control outbreaks but is difficult to sustain as a long-term control measure because rodents from surrounding fields and forests will likely re-colonize the village after a short time.

Considerable effort has been put into combating Lassa fever in Kenema District over the years through the work of the Sierra Leone Ministry of Health and Sanitation with support from various international partners, primarily through programs based at Kenema Government Hospital (Figure 1) [23]. Activities include the enhancement of laboratory diagnostic capacity, training in laboratory diagnosis, clinical management, and infection and environmental control. An outreach team conducts follow-up investigations at the house of each confirmed case, including contact tracing and an environmental assessment, and makes recommendations for improving the area hygiene to discourage rodent colonization. A zoology team often traps rodents in and around the house for 1–2 nights. The outreach team also conducts periodic health education campaigns in susceptible communities.

Despite the considerable and laudable control efforts described above, there is no evidence that the incidence of Lassa fever in Kenema District is decreasing. In fact, according to Sierra Leone Ministry of Health and Sanitation statistics, the number of reported cases of the disease in Kenema has increased every year since 2004 and there is evidence, still to be confirmed, that the endemic area may be expanding to other areas of Sierra Leone based on increasing observation of cases in areas where none had previously been seen [24]. Furthermore, although we recognize the potential importance of measures such as health education campaigns and improved village hygiene in controlling Lassa fever, economic and logistical barriers may make these measures more complicated than they first appear; even the resources to reliably and consistently cover food stuffs may be difficult to maintain, recalling that, despite its diamonds, Kenema District, particularly the regions where Lassa fever is highly endemic, is one the poorest districts in one of the poorest countries in the world. Ultimately, these measures fail to directly address the biggest underlying risk factor—poor quality housing. Any long-term solution to the problem of Lassa fever in Kenema District must address this root problem.

### Housing policy in Sierra Leone

Throughout most of the 1900s neither the colonial or independent governments of Sierra Leone were directly involved in the provision of housing, preferring to leave the matter to the private sector [25]. Prior to 2005, laws developed in 1946 and 1960 were the last pieces of legislation that the Government of Sierra Leone had passed for housing and regional/urban planning. Most policies and laws were oriented more toward issues of land tenure rather than the structures built on the land, with the exceptions almost uniformly being directed at problems of crowding and public health in the capitol, Freetown.

The onset of civil war in 1991 necessitated a change in priority to measures related to emergency shelter as opposed to longer-term development. Since the war, new policies to combat high rates of urbanization in Freetown and resettle war victims and displaced individuals have been the focus of the Ministry of Lands, Country Planning and the Environment. Some progress has been made; in 2006 a partnership between the government of Sierra Leone, UN-HABITAT, and the UNDP resulted in a revised and updated national housing policy and an outline of a national housing program [26]. However, further work on this program has been placed on hold until the issue of land tenure is fully addressed. Likewise, in the 2005 Land (Acquisition and Commercial Use) Bill, issues of land tenure were prioritized over housing [27].

While the Ministry of Lands, Country Planning and the Environment moves to develop a national land policy, the Sierra Leone National Social Security and Insurance Trust piloted an affordable housing project for low-income earners in four provincial district capital cities, including Kenema [28]. The first stage of this project culminated in 2010 with six new houses with high-quality foundations built as a private option. This is a success story for the housing business, but the measure generally only applies to civil servants or others formally employed and paying social security, which is not generally the case for most of those living in poor rural communities where the risk of Lassa fever is highest. Sierra Leone needs a cost-effective public option to provide shelter and prevent disease in the most destitute portion of its population.

### Right to housing as a means of right to health and prevention of Lassa fever: a proposed step forward for housing and health equity

As Sierra Leoneans in Kenema District rebuild their lives and homes in the post-war era, they continue to face increased risk of Lassa fever, as well as the many other risks and stressors to physical and mental health that come with inadequate shelter. The prevention of Lassa fever can be viewed as a human rights struggle, with the provision of acceptable quality housing as a major component of the solution. Clearly, assistance is needed to address this basic human right. The Government of Sierra Leone has a responsibility to assist in the development of quality housing for its citizens, especially given the evidence linking housing quality and risk of Lassa fever. However, we recognize that there are significant real-world economic challenges to this task; it is relatively easy to agree on the human rights to which we are all entitled, but much more difficult to determine from where the resources must come to provide those rights.

As a first step toward a solution, we propose a pilot program to promote the development of quality housing and diminish the risk of Lassa fever in Kenema District. In such a program, the Government of Sierra Leone might first work in partnership with international and non-governmental organizations with expertise in housing design, such as UN-HABITAT, Habitat for Humanity, and Architects Without Borders, to develop an architectural plan for low-cost houses specifically designed to limit rodent invasion, ideally using locally available materials. The design might involve new stand-alone houses or modifications to existing structures to impede rodent entry, such as reinforcing floors and walls to prevent rodents from burrowing. The government would then provide raw materials and logistic support to community members who want to implement the housing improvements. Ideally the materials would be free, but other options would be to make them available at a subsidized cost or under favorable loan conditions (cash or commodity). Utilization of local resources and labor for production of building materials would enhance local economies, establish supply chains, and ensure sustainability after donor funding is gone. The housing structural changes would be added to components of education, rodent control, and improved community hygiene as part of an integrated pest management program [29]. Monitoring of rodent abundance and incidence of Lassa fever in and outside areas of program implementation could be used to objectively assess the program’s efficacy and guide further implementation. One or more of the various research organizations working in Kenema District could be engaged to assist with monitoring and evaluation [23]. If successful, the program could be expanded to other districts of Sierra Leone and even other countries.

We recognize that interventions incorporating economic strategies add significant initial expense to public health programs that low-to-middle income countries cannot easily afford. Proper housing is not free and the proposed pilot project would entail significant economic challenges to the Government of Sierra Leone. Nevertheless, incorporating economic components into public health interventions is increasingly recognized as essential for effectiveness across disciplines of global health. In the world of women’s rights, for example, interventions with economic strategies, such as incentive-based monthly allowances to families in Malawi and Tanzania or microfinance programs in South Africa, reduced incidence of HIV infection in young women beyond more traditional knowledge- and behavioral-based interventions [30,31].

Although a detailed cost-effectiveness analysis of the proposed housing program would have to be conducted, we suspect that the decrease in incidence of Lassa fever and enhanced general well-being from improved housing equity would likely ultimately offset the cost of the program, especially considering that most healthcare in Sierra Leone is provided by the public sector, with limited cost recovery [32]. Such a program would also protect from other rodent-borne diseases, such as leptospirosis, plague, and leishmaniasis. Expansion of the program to provide door and window screens to prevent malaria, the most common vector-borne disease in Sierra Leone, could further improve health and diminish expensive healthcare outlays by the government. Looking further downstream, a healthier population is, of course, more productive in the work-force, helping to expand the fragile post-war economy in Sierra Leone.

## Competing interest

The authors declare that they have no competing interests.

## Authors’ contributions

All authors contributed equally to conceptualize and write this manuscript.

## Pre-publication history

The pre-publication history for this paper can be accessed here:

http://www.biomedcentral.com/1472-698X/13/2/prepub

## References

[B1] Enria DA Mills JN Bausch D Shieh W Peters CJ Guerrant RL, Walker DH, Weller PF Arenavirus Infections Tropical Infectious Diseases: Principles, Pathogens, and Practice 2011 3rd Philadelphia: Churchill Livingstone 449 461

[B2] McCormick JB Webb PA Krebs JW Johnson KM Smith ES A prospective study of the epidemiology and ecology of Lassa fever J Infect Dis 1987 155 3 437 444 10.1093/infdis/155.3.437 3805771

[B3] Richmond JK Baglole DJ Lassa fever: epidemiology, clinical features, and social consequences BMJ 2003 327 7426 1271 1275 10.1136/bmj.327.7426.1271 14644972 PMC286250

[B4] Fichet-Calvet E Rogers DJ Risk maps of Lassa fever in West Africa PLoS Negl Trop Dis 2009 3 3 e388 10.1371/journal.pntd.0000388 19255625 PMC2644764

[B5] Ehichioya DU Hass M Olschlager S Becker-Ziaja B Onyebuchi Chukwu CO Coker J Nasidi A Ogugua OO Gunther S Omilabu SA Lassa fever, Nigeria, 2005–2008 Emerg Infect Dis 2010 16 6 1040 1041 10.3201/eid1606.100080 20507773 PMC3086228

[B6] Monath TP Newhouse VF Kemp GE Setzer HW Cacciapuoti A Lassa virus isolation from Mastomys natalensis rodents during an epidemic in Sierra Leone Science 1974 185 147 263 265 10.1126/science.185.4147.263 4833828

[B7] Katakweba AAS Mulungu LS Eiseb SJ Mahlaba TA Makundi RH Massawe AW Borremans B Belmain SR Prevalence of haemoparasites, leptospires and coccobacilli with potential for human infection in the blood of rodents and shrews from selected localities in Tanzania, Namibia and Swaziland BioOne 2012 47 1 119 127

[B8] Mgode GF Mhamphi G Katakweba A Paemelaere E Willekens N Leirs H Machang’u RS Hartskeerl RA PCR detection of *Leptospira* DNA in rodents and insectivores from Tanzania Belg J Zool 2005 135 suppl 17 19

[B9] Green CA Gordon DH Lyons NF Biological species in Praomys (Mastomys) natalensis (Smith), a rodent carrier of Lassa virus and bubonic plague in Africa Am J Trop Med Hyg 1978 27 3 627 629 677375 10.4269/ajtmh.1978.27.627

[B10] Githure JI Schnur LF Le Blancq SM Hendricks LD Characterization of Kenyan Leishmania spp. and identification of Mastomys natalensis, Taterillus emini and Aethomys kaiseri as new hosts of Leishmania major Ann Trop Med Parasitol 1986 80 5 501 507 3632097 10.1080/00034983.1986.11812056

[B11] Ikeh EI Ajayi JA Nwana EJ Mastomys natalensis and Tatera gambiana as probable reservoirs of human cutaneous leishmaniasis in Nigeria Trans R Soc Trop Med Hyg 1995 89 1 25 26 10.1016/0035-9203(95)90642-8 7747299

[B12] Keenlyside RA McCormick JB Webb PA Smith E Elliott L Johnson KM Case–control study of Mastomys natalensis and humans in Lassa virus-infected households in Sierra Leone Am J Trop Med Hyg 1983 32 4 829 837 6881432 10.4269/ajtmh.1983.32.829

[B13] Demby AH Inapogui A Kargbo K Koninga J Kourouma K Kanu J Coulibaly M Wagoner KD Ksiazek TG Peters CJ Lassa fever in Guinea: II. Distribution and prevalence of Lassa virus infection in small mammals Vector Borne Zoonotic Dis 2001 1 4 283 297 10.1089/15303660160025912 12653128

[B14] Safronetz D Lopez JE Sogoba N Traore SF Raffel SJ Fischer ER Ebihara H Branco L Garry RF Schwan TG Detection of Lassa virus, Mali Emerg Infect Dis 2010 16 7 1123 1126 10.3201/eid1607.100146 20587185 PMC3321918

[B15] Lecompte E Fichet-Calvet E Daffis S Koulemou K Sylla O Kourouma F Dore A Soropogui B Aniskin V Allali B Mastomys natalensis and Lassa fever, West Africa Emerg Infect Dis 2006 12 12 1971 1974 10.3201/eid1212.060812 17326956 PMC3291371

[B16] Ter Meulen J Lukashevich I Sidibe K Inapogui A Marx M Dorlemann A Yansane ML Koulemou K Chang-Claude J Schmitz H Hunting of peridomestic rodents and consumption of their meat as possible risk factors for rodent-to-human transmission of Lassa virus in the Republic of Guinea Am J Trop Med Hyg 1996 55 6 661 666 9025695 10.4269/ajtmh.1996.55.661

[B17] Fraser DW Campbell CC Monath TP Goff PA Gregg MB Lassa fever in the Eastern Province of Sierra Leone, 1970–1972. I. Epidemiologic studies Am J Trop Med Hyg 1974 23 6 1131 1139 4429182 10.4269/ajtmh.1974.23.1131

[B18] McCormick JB King IJ Webb PA Johnson KM O'Sullivan R Smith ES Trippel S Tong TC A case–control study of the clinical diagnosis and course of Lassa fever J Infect Dis 1987 155 3 445 455 10.1093/infdis/155.3.445 3805772

[B19] United Nations Development Programme Sierra Leone Human Development Report: Empowering Local Government for Sustainable Development and Poverty Reduction 2007

[B20] Winnebah T Brewah F Francis T 2004 Population and Housing Census: Analytical Report on Poverty. Republic of Sierra Leone Republic of Sierra Leone 2006 2006 1 55

[B21] Bonner PC Schmidt WP Belmain SR Oshin B Baglole D Borchert M Poor housing quality increases risk of rodent infestation and Lassa fever in refugee camps of Sierra Leone Am J Trop Med Hyg 2007 77 1 169 175 17620650

[B22] Moses L Kargbo K Koninga J Robert W Lungay V Fonnie R Kanneh L Bangura J Garry R Bausch D Household predictors of abundance of the Lassa virus reservoir, Mastomys natalensis, in the Eastern Province of Sierra Leone. Abstract, 58th annual meeting of the American Society of Tropical Medicine and Hygiene 2009 Washington, DC

[B23] Khan S Goba A Chu M Roth C Healing T Marx A Fair J Guttieri M Ferro P Imes T New opportunities for field research on the pathogenesis and treatment of Lassa fever Antiviral Res 2008 78 1 103 115 10.1016/j.antiviral.2007.11.003 18241935

[B24] Lassa Fever-Sierra Leone (03): (Northern) Promed-mail: 20101017.3770, Oct. 17, 2010. Available at: http://www.promedmail.org/direct.php?id=20101017.3770

[B25] Doherty J Housing and development in Freetown, Sierra Leone Cities 1985 2 2 149 164 10.1016/0264-2751(85)90116-7

[B26] National Housing Policy and Development Programme–Sierra Leone http://www.unhabitat.org/content.asp?cid=4609&catid=232&typeid=13&subMenuId=0

[B27] Renner-Thomas A Land tenure in Sierra Leone: the law, dualism, and the making of a land policy 2010 Central Milton Keynes: AuthorHouse UK Ltd.

[B28] Renner-Thomas A In Sierra Leone, NASSIT Solves Sierra Leone's Housing Crises 2010 Awareness Times Available at: http://news.sl/drwebsite/exec/view.cgi?archive=6&num=15969&printer=1

[B29] Integrated Pest Management (IPM) Principles Available at: http://www.epa.gov/opp00001/factsheets/ipm.htm

[B30] A Cash Transfer Program Reduces HIV Infections among Adolescent Girls. Malawi and Tanzania Research Shows Promise in Preventing HIV and Sexually-Transmitted Infections Development Research Group of the World Bank July 18, 2010. Available at: http://web.worldbank.org/WBSITE/EXTERNAL/COUNTRIES/AFRICAEXT/EXTAFRHEANUTPOP/EXTAFRREGTOPHIVAIDS/0,,contentMDK:22649337~menuPK:717155~pagePK:34004173~piPK:34003707~theSitePK:717148,00.html

[B31] Jewkes R Nduna M Levin J Jama N Dunkle K Khuzwayo N Koss M Puren A Wood K Duvvury N A cluster randomized-controlled trial to determine the effectiveness of Stepping Stones in preventing HIV infections and promoting safer sexual behaviour amongst youth in the rural Eastern Cape, South Africa: trial design, methods and baseline findings Trop Med Int Health 2006 11 1 3 16 10.1111/j.1365-3156.2005.01530.x 16398750

[B32] Donnelly J How did Sierra Leone provide free health care? Lancet 2011 377 9775 1393 1396 10.1016/S0140-6736(11)60559-X 21520507

